# Bone morphogenetic protein 6–mediated crosstalk between endothelial cells and hepatocytes recapitulates the iron-sensing pathway *in vitro*

**DOI:** 10.1016/j.jbc.2021.101378

**Published:** 2021-11-02

**Authors:** Shijin Wang, Cheng Chen, Linna Yu, Johannes Mueller, Vanessa Rausch, Sebastian Mueller

**Affiliations:** Center for Alcohol Research and Salem Medical Center, University of Heidelberg, Heidelberg, Germany

**Keywords:** endothelial cells, BMP6–SMAD pathway, hepatocytes, hepcidin, iron metabolism, ALK, ALK receptor tyrosine kinase, BMP6, bone morphogenetic protein 6, BMPER, BMP-binding endothelial regulator, CM, conditioned medium, EC, endothelial cell, ECGM, endothelial cell growth medium, FAC, ferric ammonium citrate, FCS, fetal calf serum, hemin, ferric chloride heme, HJV, hemojuvelin, HUVEC, human umbilical vein endothelial cell, Id1, inhibitor of DNA binding 1, LDN, LDN193189 hydrochloride, LSEC, liver sinusoidal endothelial cell, pSMAD1/5/8, phosphorylated small mothers against decapentaplegic 1,5,8, qRT-PCR, real-time quantitative PCR, SFG, sodium ferric gluconate, SMAD1, small mothers against decapentaplegic homolog 1, STAT3, signal transducer and activator of transcription 3

## Abstract

Liver sinusoidal endothelial cell–derived bone morphogenetic protein 6 (BMP6) and the BMP6–small mothers against decapentaplegic homolog (SMAD) signaling pathway are essential for the expression of hepcidin, the secretion of which is considered the systemic master switch of iron homeostasis. However, there are continued controversies related to the strong and direct suppressive effect of iron on hepatocellular hepcidin *in vitro* in contrast to *in vivo* conditions. Here, we directly studied the crosstalk between endothelial cells (ECs) and hepatocytes using *in vitro* coculture models that mimic hepcidin signaling *in vivo*. Huh7 cells were directly cocultured with ECs, and EC conditioned media (CM) were also used to culture Huh7 cells and primary mouse hepatocytes. To explore the reactions of ECs to surrounding iron, they were grown in the presence of ferric ammonium citrate and heme, two iron-containing molecules. We found that both direct coculture with ECs and EC-CM significantly increased hepcidin expression in Huh7 cells. The upstream SMAD pathway, including phosphorylated SMAD1/5/8, SMAD1, and inhibitor of DNA binding 1, was induced by EC-CM, promoting hepcidin expression. Efficient blockage of this EC-mediated hepcidin upregulation by an inhibitor of the BMP6 receptor ALK receptor tyrosine kinase 2/3 or BMP6 siRNA identified BMP6 as a major hepcidin regulator in this coculture system, which highly fits the model of hepcidin regulation by iron *in vivo*. In addition, EC-derived BMP6 and hepcidin were highly sensitive to levels of not only ferric iron but also heme as low as 500 nM. We here establish a hepatocyte–endothelial coculture system to fully recapitulate iron regulation by hepcidin using EC-derived BMP6.

Iron is essential to the metabolism of all cells but also highly toxic in the light of Fenton chemistry (1). To maintain adequate and safe levels of iron, a complex and refined regulatory machinery has evolved that tightly controls both intracellular and systemic iron metabolism (2). In mammals, more than half of the total iron is contained in the oxygen carrier hemoglobin, while liver ferritin forms another important iron reservoir of ca. 20%. Systemic iron levels are mainly controlled by the iron master switch hepcidin that coordinates the continuous release of iron from reticuloendothelial macrophages through recycled heme (3, 4) and, to a minor extent, by duodenal iron absorption. Hepcidin is primarily expressed in hepatocytes as a precursor propeptide and to a lesser extent in some other cell types such as macrophages and cardiomyocytes (5). It is mainly regulated at the transcription level, and mRNA levels correspond well with concentrations of the peptide (6). By binding to and degrading the iron exporter ferroportin 1, which is localized at the plasma membrane of duodenal enterocytes, macrophages, and hepatocytes, hepcidin efficiently blocks iron absorption and iron recycling (7). Consequently, its overexpression leads to hypoferremia and anemia (8), whereas suppression of hepcidin causes iron overload (9).

The regulation of hepcidin is complex, and the direct mechanisms of iron sensing are still not completely understood. Bone morphogenetic protein 6 (BMP6) released from endothelial cells (ECs) has been shown to efficiently induce hepcidin *via* the small mothers against decapentaplegic homolog (SMAD) pathway (10). BMP6 binds to the BMP receptor on the liver cell membrane and its coreceptor hemojuvelin (HJV), forming a complex (11) that joins type I (ALK receptor tyrosine kinase [ALK]2 and ALK3) and the type II BMP receptors to induce the phosphorylation of SMAD1/5/8. The latter interacts with SMAD4 to form the SMAD complex and then translocates into the nucleus and binds to the hepcidin promoter (12). Inhibitor of DNA binding 1 (Id1), protein atonal homolog 8, and inhibitory Smad6 and Smad7 are other important modulators of the BMP–SMAD pathway (13, 14). In addition, typical mediators of inflammation (*e.g.*, IL-6, IL-1β, hypoxia or ROS/H_2_O_2_) significantly induce hepcidin by promoting the phosphorylation of signal transducer and activator of transcription 3 (STAT3) to initiate STAT3-mediated hepcidin signaling (15, 16).

Although various upstream regulators of hepcidin have been discovered (such as C/EBPα, BMP6, SMAD1/5/8, SMAD4, SMAD6, SMAD7, TMPRSS6, IL-6, CREBH, CHOP, and TLR4), the overall regulation, namely the exact sensing of iron by hepcidin, is still far from being understood. Two major experimental and clinical observations are especially difficult to understand. Although hepcidin suppression has been recognized as major mechanisms of chronic iron accumulation in most hereditary iron overload diseases such as hemochromatosis (17), hepcidin is seemingly paradoxically and profoundly suppressed in hemolytic diseases such as thalassemia (18) or chronic liver diseases (19) in the presence of iron excess, for example, released from an increased red blood cell turnover (19, 20). Another major unresolved observation is that hepcidin responds differently to iron overload *in vitro* and *in vivo* (21, 22). In contrast to the profound induction of hepcidin by iron under *in vivo* conditions, hepcidin expression is typically inhibited in the presence of iron in cell culture models (22). In a more detailed *in vitro* study, we could recently show that iron directly blocks hepatocellular hepcidin signaling through the SMAD pathway independent of STAT3 (23).

The complexity of iron sensing by hepcidin could be resolved by neither further mechanistic insights from intercellular communication between hepatocytes and ECs or macrophages (15, 24, 25) nor additional mechanistic insights of known or novel molecules such as TfR1, ERFE, or GDF15 (26, 27, 28). An alternative explanation for the paradox response of hepatocellular hepcidin to direct iron exposure could be the fact that the actual location of “iron sensing” occurs in a different type of cells, for example, in the endothelial bed, which is then indirectly communicated to hepatocytes by intercellular signaling through the BMP6–SMAD pathway. As mentioned above, BMP6 has been identified as one of the most potent upstream inducers of hepcidin (10, 29). BMP6 KO mice exhibited hepcidin deficiency and severe tissue iron overload (10, 30), although BMP2 and BMP4 can also bind to HJV (31).

Recently, a study demonstrated that liver sinusoidal endothelial cells (LSECs) are the predominant source of basal BMP6 secretion and participate in iron-regulated hepcidin expression (25). Although the role of both LSECs and ECs in systemic iron regulation has been only marginally studied, a few studies on the blood–brain barrier and retina suggest that they may be involved in iron sensing and transport (32, 33). Moreover, ECs are highly sensitive to iron changes (34, 35, 36) and, finally, they secrete the autocrine protein BMP-binding endothelial regulator (BMPER), able to antagonize BMP activity and regulate hepcidin (37). Finally, a recent study provided the first insights into molecular iron sensing by ECs. Accordingly, Nrf2 is activated by iron-induced, mitochondria-derived pro-oxidants and drives BMP6 expression in LSECs (38).

In the present study, we establish a coculture system of ECs and hepatocytes to study the crosstalk between ECs and hepatocytes with regard to iron regulation *in vitro*, mainly how the EC-derived BMP6 affects hepatocellular hepcidin and their response to iron. Our data suggest that an EC–hepatocyte coculture system suffices to recapitulate iron sensing as observed *in vivo*.

## Results

### ECs strongly enhance hepcidin in liver cells

**Figure 1:**
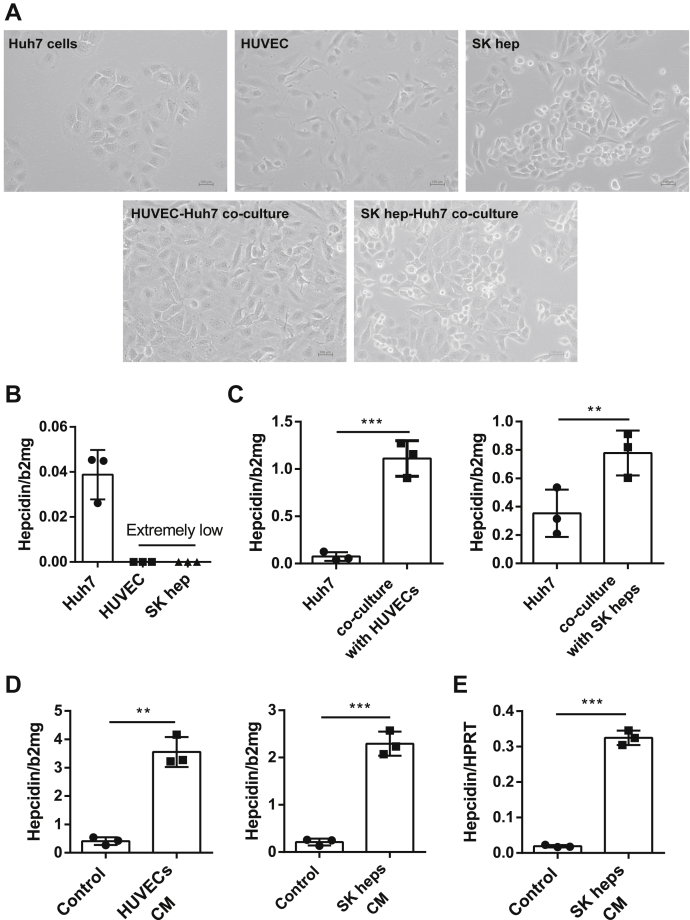
**Endothelial cells strongly increase hepcidin in liver cells.***A*, Huh7 cells were cultured with HUVECs with a ratio of 1:1 for 24 h in the endothelial cell growth medium (ECGM) with 2% fetal calf serum (FCS) or cocultured with SK heps in Dulbecco's modified Eagle's medium (DMEM) with 2% FCS. Huh7 cell monolayer culture under the same media was used as the control. *B*, Huh7 cells have ca. 1000 times higher hepcidin mRNA levels than the two endothelial cell lines, HUVEC and SK hep. Absolute hepcidin expressions were determined by qRT-PCR and calculated by the following formula: (efficiency of reference gene)ˆCq/(efficiency of target gene)ˆCq. *C*, Huh7 cells directly cocultured with endothelial HUVECs (cell ratio 1:1) or SK heps (cell ratio 1:1) for 24 h showed significantly increasing hepcidin mRNA expression compared with Huh7 cell monoculture. Data are representative of three independent experiments (n = 3). *D*, ECGM with 2% FCS on HUVECs or DMEM with 2% FCS on SK heps was collected after 24 h of culturing and was immediately put on Huh7 cells. Huh7 cells cultured with HUVEC–conditioned medium (CM) or with SK hep–CM for 24 h expressed much more hepcidin than Huh7 cells cultured by the same kinds but unconditioned media. Data are representative of three independent experiments (n = 3). *E*, primary murine hepatocytes were isolated from 8-week-old male mice with C57BL/6 background. They were seeded in standard Williams' medium for attachment for 24 h and then incubated with SK hep–CM for 24 h. Primary hepatocytes under SK hep–CM had higher hepcidin transcription levels than the control cells under unconditioned medium. Target mRNA expression was determined by qRT-PCR. PCR results were normalized to hypoxanthine guanine phosphoribosyltransferase (HPRT) or beta-2-microglobulin (β2MG). Data are presented as dot plots with the mean ± SD, and significant differences are marked by *asterisks* (∗∗*p* < 0.01; ∗∗∗*p* < 0.001). HUVECs, human umbilical vein endothelial cells; qRT-PCR, real-time quantitative PCR.

We used two established EC lines to study the crosstalk between ECs and Huh7 cells in detail. Human umbilical vein endothelial cells (HUVECs) are isolated from human umbilical veins, and they are widely used as a normal endothelial cell line (39, 40). SK hep cells are originally derived from human liver adenocarcinoma and morphologically resemble LSECs (41). Two different *in vitro* coculture systems were established. A two-dimensional coculture system of ECs and Huh7 cells was established by seeding Huh7 cells first for 24 h and adding HUVECs or SK heps on top thereafter (Fig. 1*A*). The cell ratio between Huh7 and ECs was 1:1. Huh7 cell monoculture served as the control for the direct coculture systems. In a second indirect approach, conditioned media (CM) were obtained from HUVECs (in complete endothelial cell growth medium [ECGM] containing 2% fetal calf serum [FCS]) or SK heps (in Dulbecco's modified Eagle's medium [DMEM] containing 2% FCS) monolayer culture after 24 h, and fresh ECGM or DMEM with 2% FCS served as controls. As shown in Figure 1*B*, hepcidin mRNA from such an Huh7–EC coculture is almost exclusively derived from Huh7 cells (>99.9%). Huh7 cells directly cocultured with HUVECs or SK hep showed significantly higher hepcidin expression than Huh7 cell monoculture (Fig. 1*C*). Moreover, EC-CM by both HUVEC and SK hep cells drastically induced hepcidin as compared with Huh7 cells alone in fresh control media (Fig. 1*D*). SK hep–CM also strongly induced hepcidin mRNA in murine primary hepatocytes within 24 h (Fig. 1*E*). In summary, our data demonstrate that basal expression of hepatocyte hepcidin is strongly induced in the presence of ECs or EC-CM.

### SMAD pathway is activated in hepatocytes by ECs

To confirm the BMP6–SMAD signaling response to iron loading *in vivo*, sodium ferric gluconate (SFG), a widely used iron donor in humans, was intravenously injected into mice. The same volume of saline served as control. After 12 h, the serum iron concentration of the SFG group significantly increased to 74 μmol/l, which was twice as compared with the control group (Fig. S1*A*). Iron injection caused both an increase of BMP6 and hepcidin mRNA expression in the mouse liver by almost three times (Fig. S1, *B* and *C*), whereas BMP2 mRNA expression in the murine liver was not statistically changed (Fig. S1*D*). In addition, according to the reported important role of BMP6 in hepcidin regulation, we next directly treated human hepatoma cells Huh7 with recombinant human BMP6 protein. After 24 h, hepcidin mRNA and the phosphorylated SMAD1/5/8 (pSMAD1/5/8) protein in Huh7 cells under BMP6 were highly increased (Fig. S1, *E* and *F*).

**Figure 2:**
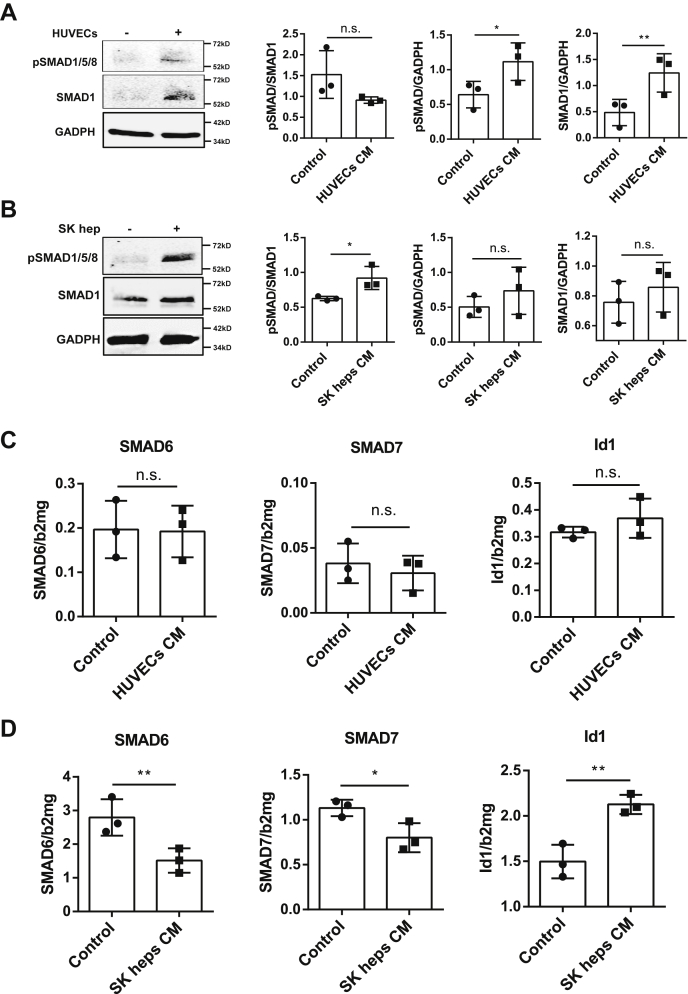
**The SMAD pathway is activated in hepatocytes by endothelial cell–conditioned media.***A*, HUVECs conditioned medium (CM) promoted pSMAD1/5/8 and SMAD1 total protein (normalized to GADPH protein levels) simultaneously in Huh7 cells in 24 h. The pSMAD-SMAD protein ratio was not significantly changed by HUVEC-CM. Representative data of three independent experiments are used for statistical analysis. *B*, SK hep–CM promoted pSMAD-SMAD1 ratio in Huh7 cells after 24 h, whereas unconditioned medium had no significant effect on pSMAD–SMAD. Normalized pSMAD1/5/8 or SMAD1 protein levels were not statistically changed by HUVEC-CM. Representative data of three independent experiments are used for statistical analysis. *C*, there were no significant transcriptional changes of the inhibitory molecules of the SMAD pathway such as SMAD6, SMAD7, or Id1 in Huh7 cells cultured by HUVEC-CM for 24 h. Representative data of three independent experiments (n = 3 of each time) are shown. *D*, SMAD6 and SMAD7 mRNA expression was inhibited in Huh7 cells under SK hep–CM for 24 h, and the Id1 mRNA expression was increased. The HUVEC-CM used in these experiments was the endothelial cell growth medium (ECGM) containing 2% fetal calf serum (FCS) from 24-h culture of HUVECs, and the SK hep–CM here was the Dulbecco's modified Eagle's medium (DMEM) containing 2% FCS from 24-h culture with SK heps. Representative data of three independent experiments (n = 3 of each time) are shown. Transcriptional changes of target genes were determined by qRT-PCR, and the results were normalized to beta-2-microglobulin (β2MG). Data are presented as dot plots with the mean ± SD, and significant differences are marked by *asterisks* (∗*p* < 0.05; ∗∗*p* < 0.01). HUVECs, human umbilical vein endothelial cells; n.s., not significant; pSMAD1/5/8, phosphorylated SMAD1/5/8; qRT-PCR, real-time quantitative PCR; SMAD, small mothers against decapentaplegic homolog.

Because the BMP6–SMAD signaling pathway is considered essential for iron-mediated hepcidin regulation, we studied in more detail SMAD signaling in our EC–Huh7 coculture systems. In Figure 2*A*, both pSMAD1/5/8 protein and SMAD1 total protein were upregulated in the presence of HUVECs-CM, although the pSMAD-SMAD1 ratio was not significantly altered. In contrast to the experiments with HUVEC-CM, coculture with SK heps–CM showed increased pSMAD-SMAD1 ratio, whereas total protein amounts (normalized to GADPH) of either pSMAD1/5/8 or SMAD1 were not significantly changed (Fig. 2*B*). Consistently, Huh7 expression of SMAD6, SMAD7, and Id1 mRNA cells was also not statistically altered by HUVEC-CM (Fig. 2*C*), whereas inhibitory SMADs 6 and 7 mRNA were decreased and Id1 mRNA was increased by SK heps–CM (Fig. 2*D*). Taken together, these results indicate that SMAD signaling of Huh7 cells is activated by ECs, especially by liver-specific EC–SK heps.

### Silencing of endothelial BMP6 blocks hepatocellular hepcidin

**Figure 3:**
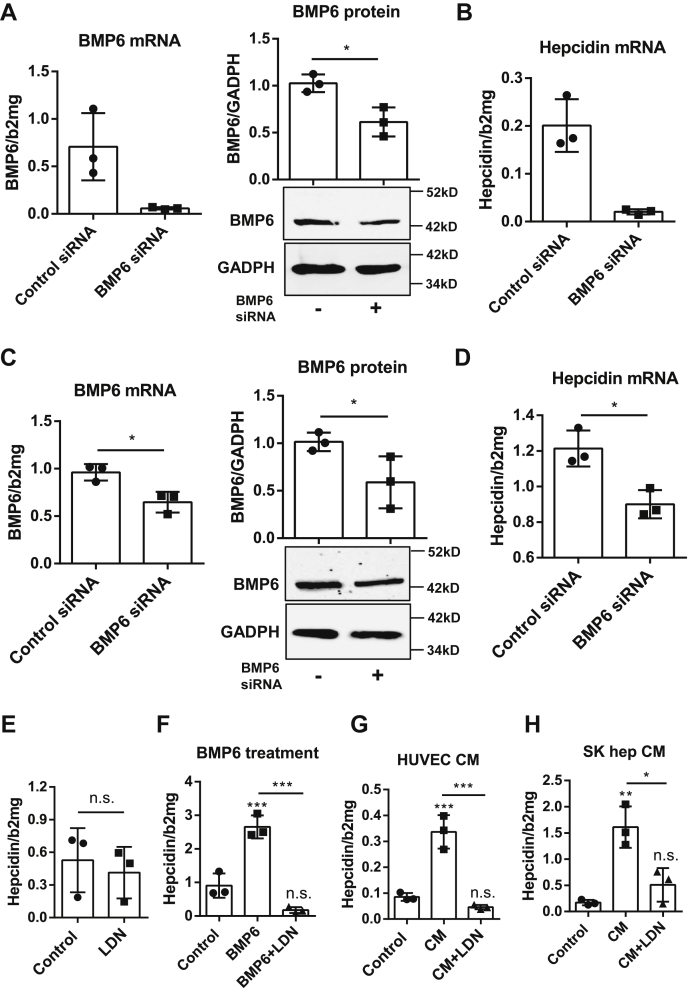
**Silencing endothelial BMP6 blocks hepatocellular hepcidin induction.***A*, BMP6 siRNA (50 nM/well) and negative siRNA (50 nM/well) as control were separately transfected into HUVECs by Lipofectamine 2000 (3 μl/well). All reagents were removed and replaced by fresh endothelial cell growth medium (EGCM) after 6 h. Knockdown efficiency was validated at 48 h by Western blot and qRT-PCR. BMP6 expression was significantly suppressed by BMP6 siRNA compared with the nontargeting control siRNA on both mRNA and protein levels. Representative data of three independent experiments (n = 3) are shown. *B*, after transfection reagents were removed and HUVECs recovered stable in fresh ECGM for 18 h, Huh7 cells (in DMEM with 10% FCS) were directly seeded on top of these transfected HUVECs (cell ratio 1:1) with Dulbecco's modified Eagle's medium (DMEM) containing 10% FCS. The seeding medium was removed at 48 h after Huh7 cells were well attached, and just fresh ECGM with 2% FCS was added to the coculture system. All cells were collected together at 72 h. In the HUVEC and Huh7 coculture system, the induction of hepatocellular hepcidin was drastically impaired by BMP6 siRNA interference in HUVECs. Representative data are shown for three independent experiments. *C*, the protocols of BMP6 siRNA transfection in SK hep and coculture with Huh7 cells were the same as those used for HUVECs except DMEM containing 2% FCS for SK heps culture. BMP6 transcription and protein levels were reduced in SK hep by BMP6 siRNA as well. Representative data are shown for three independent experiments. *D*, BMP6 silencing inhibited the hepatocellular hepcidin expression in a direct SK hep/Huh7 coculture system. Representative data are shown for three independent experiments. *E* and *F*, the hepcidin induction in Huh7 cells by BMP6 (50 ng/ml) was completely blocked by the ALK2/3 inhibitor (LDN 20 nM) after 24 h of treatment, whereas the LDN did not significantly affect basal hepcidin expression. This experiment on Huh7 cells was operated under normal medium for Huh7 culture (DMEM with 10% FCS). *G*, ALK2/3 inhibitor (LDN 20 nM) in HUVEC conditioned medium (CM) decreased hepcidin expression in Huh7 after 24 h. Representative data are from three independent experiments (n = 3). *H*, hepcidin transcription level was inhibited by the ALK2/3 inhibitor (LDN 20 nM) as well. Representative data of three independent experiments (n = 3 of each time) are used for statistical analysis. Target proteins were determined by Western blot and normalized to GADPH. Transcriptional changes of target genes were determined by qRT-PCR, and the results were normalized to beta-2-microglobulin (β2MG) or hypoxanthine guanine phosphoribosyltransferase (HPRT). Data are presented as dot plots with the mean ± SD, and significant differences are marked by *asterisks* (∗*p* < 0.05; ∗∗*p* < 0.01; ∗∗∗*p* < 0.001). ALK, ALK receptor tyrosine kinase; BMP6, Bone morphogenetic protein 6; HUVECs, human umbilical vein endothelial cells; FCS, fetal calf serum; LDN, LDN193189 hydrochloride; n.s., not significant; qRT-PCR, real-time quantitative PCR.

We next set up a series of experiments to identify the primary EC-derived factor that induces hepcidin. Considering the significant increase of BMP6 but not BMP2 by iron injection *in vivo*, we primarily focused on the role of endothelial-derived BMP6. First, we applied BMP6 siRNA to HUVEC or SK hep to effectively block BMP6 production. We also checked for BMP2 expression but found no significant alteration in either HUVECs or SK heps (Fig. S2, *A* and *B*). After removing the siRNA and washing, Huh7 cells were seeded on top of HUVECs or SK hep for observing hepcidin changes in coculture systems. BMP6 siRNA significantly deleted BMP6 mRNA (knockdown efficiency was around 80%) and also lessened protein expression in HUVECs (Fig. 3*A*), and consequently, no hepcidin activation was observed in the direct coculture with huh7 cells (Fig. 3*B*). Compared with siRNA transfection in HUVECs, the impact of siRNA on SK hep BMP6 was less pronounced (knockdown efficiency around 40%) (Fig. 3*C*) and causing smaller decrease of hepcidin during coculture, but it was still able to alleviate the induction of hepcidin in the direct coculture system (Fig. 3*D*).

Second, the ALK2/3 inhibitor LDN193189 hydrochloride (LDN) completely blocked BMP6-mediated hepcidin expression (Fig. 3*F*). Of note, LDN had no significant effect on hepcidin expression in the Huh7 monoculture system (Fig. 3*E*). The addition of LDN to CM significantly decreased hepcidin upregulation in Huh7 cells compared with Huh7 cells exposed to HUVEC-CM or SK heps–CM (Fig. 3, *G* and *H*). Id1 mRNA was induced by SK heps–CM but also inhibited by LDN (Fig. S3). In conclusion, using two strategies (mRNA silencing and pharmacological blockage), we demonstrate that endothelial-derived BMP6 is a major upstream regulator of hepcidin in a direct or indirect EC–hepatocyte coculture system.

### EC-derived BMP6 is rapidly secreted in response to low iron levels

**Figure 4:**
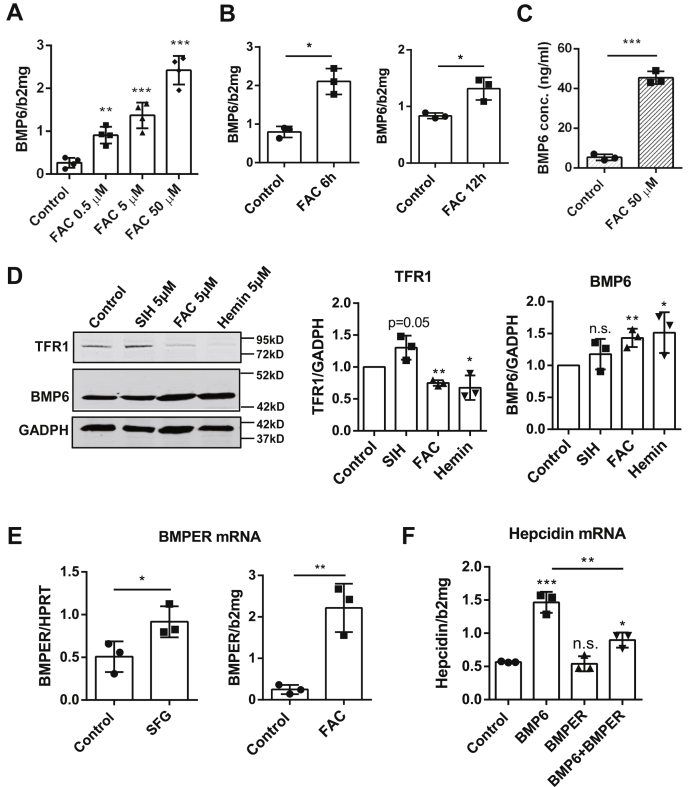
**Endothelial cells are highly responsive to surrounding ferric iron changes.***A*, concentration gradient of iron (ferric ammonium citrate [FAC] 0.5 μM, 5 μM, 50 μM in medium) gradually upregulated BMP6 mRNA expression in HUVECs. *B*, BMP6 transcription in HUVECs was upregulated by 50 μM FAC after 6 h, and this upregulation still lasted after 12 h of treatment. *C*, iron (FAC 50 μM) significantly induces BMP6 concentration in the supernatants of cultured HUVECs over a time period of 24 h as measured by human BMP6 ELISA. *D*, treatment of HUVECs with 5 μM FAC or with 5 μM hemin for 24 h could both induce BMP6 expression and inhibited transferrin receptor 1 (TFR1) expression at the protein level. TFR1 was induced in the presence of the membrane-permeable iron chelator salicylaldehyde isonicotinoyl hydrazone (SIH) (5 μM). Representative data of three independent Western blots are used for statistical analysis. *E*, endothelial-released BMPER, an autocrine BMP inhibitor, was likewise induced both *in vivo* (*left*) and *in vitro* (*right*) in response to iron at the mRNA level. Mice were iron-loaded using SFG (three mice in each group), whereas HUVECs were treated with 50 μM FAC for 24 h. *F*, human recombinant BMPER protein (50 ng/ml in medium) had no influence on basal hepcidin expression in Huh7 cells, whereas it strongly blocked the hepcidin expression in response to recombinant BMP6 (50 ng/ml). Data are shown as the mean ± SD and n = 3. Further conditions are as follows: HUVECs in panels *A*–*E* were under endothelial cell growth medium containing 2% FCS, and Huh7 cells in panel *F* were cultured with Dulbecco's modified Eagle's medium (DMEM) containing 10% FCS. The mRNA expression was determined by qRT-PCR. PCR results were normalized to hypoxanthine guanine phosphoribosyltransferase (HPRT) or beta-2-microglobulin (β2MG). Data are presented as dot plots and the mean ± SD; significant differences are marked by *asterisks* (∗*p* < 0.05; ∗∗*p* < 0.01; ∗∗∗*p* < 0.001). BMP6, Bone morphogenetic protein 6; BMPER, BMP-binding endothelial regulator; FCS, fetal calf serum; hemin, ferric chloride heme; HUVECs, human umbilical vein endothelial cells; n.s., not significant; qRT-PCR, real-time quantitative PCR; SFG, sodium ferric gluconate.

Because ECs are instrumental for an integrated physiological response of hepatocellular hepcidin to iron, we set up a set of experiments to better understand the response of EC-derived BMP6 to ferric iron levels. First, HUVECs were exposed to increasing ferric ammonium citrate (FAC) concentrations for 24 h. As shown in Figure 4*A*, BMP6 mRNA increased in response to even minuscule amounts of FAC as low as 0.5 μM, whereas BMP2 mRNA was not significantly altered (Fig. S4). Detailed kinetic analysis revealed that BMP6 induction was already observed after 6 h (Fig. 4*B*). Moreover, the release of active BMP6 protein was drastically increased in response to FAC as assessed by ELISA (Fig. 4*C*). Heme as important other physiological iron forms within the endothelial bed also induced BMP6 protein expression at micromolar concentrations (Fig. 4*D*). As expected, these iron overload conditions significantly repressed protein levels of TfR1 in HUVECs. Finally, a similar response to iron was observed with BMPER, the BMP antagonizing protein that is secreted from ECs in an autocrine fashion. Both *in vivo* and *in vitro*, BMPER transcription could be induced in response to iron loading (Fig. 4*E*). Notably, BMPER efficiently counteracted part of the BMP6-mediated hepcidin upregulation but had no effect on hepcidin in the absence of BMP6 (Fig. 4*F*). In conclusion, EC-derived BMP6 is highly responsive to iron changes *in vitro* whether in the form of ferric iron or heme. The sensitive response of EC-secreted and BMP-counteracting BMPER further suggests a delicate mechanism in ECs to fine-tune hepcidin expression in liver cells.

### EC-derived BMP6 is also induced by heme and further upregulates hepcidin

**Figure 5:**
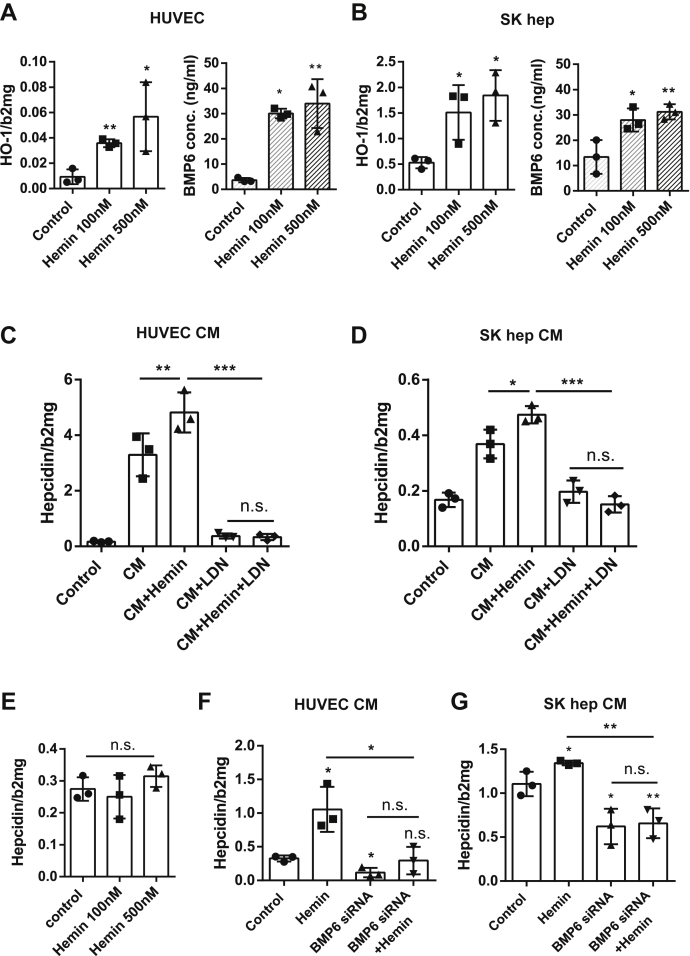
**EC-derived BMP6 is rapidly secreted in response to hemin.***A*, hemin 100 nM or 500 nM both significantly increased heme oxygenase 1 (HO-1) mRNA expression of HUVECs and BMP6 protein secretion in the culture supernatant. *B*, hemin treatments (100 nM or 500 nM) also upregulated HO-1 transcription and BMP6 protein secretion of SK hep. Representative data of three independent experiments are presented. *C*, HUVECs conditioned medium (CM) from cells preincubated with 500 nM hemin increased hepcidin expression compared with the HUVEC-CM without any pretreatment. The ALK2/3 inhibitor (20 nM LDN) blocked all the induction of hepcidin by HUVEC-CM. Data are shown as the mean ± SD and n = 3. *D*, CM from SK hep preincubated with hemin (500 nM) also increased hepcidin in Huh7 cells that could be blocked by the ALK2/3 inhibitor (LDN 20 nM). Data are shown as the mean ± SD and n = 3. *E*, hemin treatment (100 nM or 500 nM) did not significantly affect the hepcidin mRNA expression in Huh7 monocultured in fresh endothelial cell growth medium (ECGM). Representative data are from three independent experiments. *F*, Huh7 cells were seeded on top of HUVECs transfected with control siRNA or BMP6 siRNA. Transfection reagents were completely removed. After attachment, the cocultured HUVEC–Huh7 were treated with 500 nM hemin. Hemin still induced hepcidin upregulation in controls, whereas it failed to do so in BMP6-knockdown cells. Representative data are from three independent experiments. *G*, Huh7 cells were seeded on top of siBMP6-transfected SK heps. BMP6 silencing also blocked hepcidin induction by hemin in this Huh7/SK hep coculture system. Representative data of three independent experiments are presented. Cells in panels *A*, *C*, *E*, and *F* were cultured in ECGM containing 2% FCS, and cells in panels *B*, *D*, and *G* were cultured in DMEM containing 2% FCS (only Huh7 cell seeding procedures required the DMEM containing 10% FCS). BMP6 concentrations in the supernatant were measured by the direct ELISA assay. HO-1 and hepcidin mRNA expression was determined by qRT-PCR, and the results were normalized to beta-2-microglobulin (β2MG). Data are presented as dot plots with the mean ± SD, and significant differences are marked by *asterisks* (∗*p* < 0.05; ∗∗*p* < 0.01; ∗∗∗*p* < 0.001). ALK, ALK receptor tyrosine kinase; BMP6, Bone morphogenetic protein 6; ECs, endothelial cells; FCS, fetal calf serum; hemin, ferric chloride heme; HUVECs, human umbilical vein endothelial cells; LDN, LDN193189 hydrochloride; n.s., not significant; qRT-PCR, real-time quantitative PCR.

Finally, we set up a series of experiments to study EC responses to physiological iron forms such as ferric chloride heme (hemin). Because hemin is very toxic to both HUVECs and SK hep (Fig. S5, *A* and *B*), we chose concentrations of hemin as low as 100 nM and 500 nM to treat ECs. As shown in Figure 5, *A* and *B*, both HUVECs and SK hep secreted significantly more BMP6 protein under these conditions in their media. In addition, heme oxygenase 1 mRNA was also significantly increased both in SK hep and HUVECs (Fig. 5, *A* and *B*). We also confirmed that the hepcidin mRNA levels of Huh7 cells did not significantly change under the same hemin treatments using fresh EC medium (Fig. 5*E*). The CM from hemin-treated HUVECs further induced hepcidin expression compared with fresh HUVEC-CM without any hemin, and ALK2/3 inhibitor LDN could block all of the hepcidin induction by HUVEC CM with or without hemin (Fig. 5*C*). Similar results were also observed in SK hep CM (Fig. 5*D*), although they were not as pronounced as seen with HUVECs. Furthermore, 500 nM hemin was also incubated with EC–Huh7 coculture systems for 24 h after efficient silencing of BMP6 in ECs using BMP6 siRNA (for procedures, see Fig. S6). In Figure 5, *F* and *G*, a hemin-mediated increase of hepcidin was blocked by BMP6 silencing both in HUVEC–Huh7 coculture and SK hep–Huh7 coculture. In addition, hemin could not significantly induce hepcidin mRNA expression in both coculture systems after BMP6 siRNA transfection in HUVECs or SK heps (Fig. 5, *F* and *G*). In conclusion, these results demonstrate that ECs also rapidly secrete BMP6 in the presence of physiological heme, ultimately transferring into a robust hepatocellular hepcidin response.

## Discussion

We here establish an *in vitro* EC–hepatocyte coculture system that responds to iron in a BMP6-mediated fashion as is observed under *in vivo* conditions. Our study is the first to directly analyze the EC-to-hepatocytes crosstalk with regard to iron sensing and hepcidin expression. In confirmation of *in vivo* observations of strong iron-mediated induction of BMP6 and hepcidin, we found that in EC–Huh7 coculture cell models, EC-derived BMP6 not only responded to small amounts of surrounding ferric iron or hemin but was able to upregulate hepcidin in cocultured Huh7 cells through the BMP6–SMAD signaling pathway. Inhibition of hepcidin by BMP6 silencing in ECs confirmed the vital role of BMP6 for hepcidin expression and the crosstalk between ECs and liver cells.

**Figure 6:**
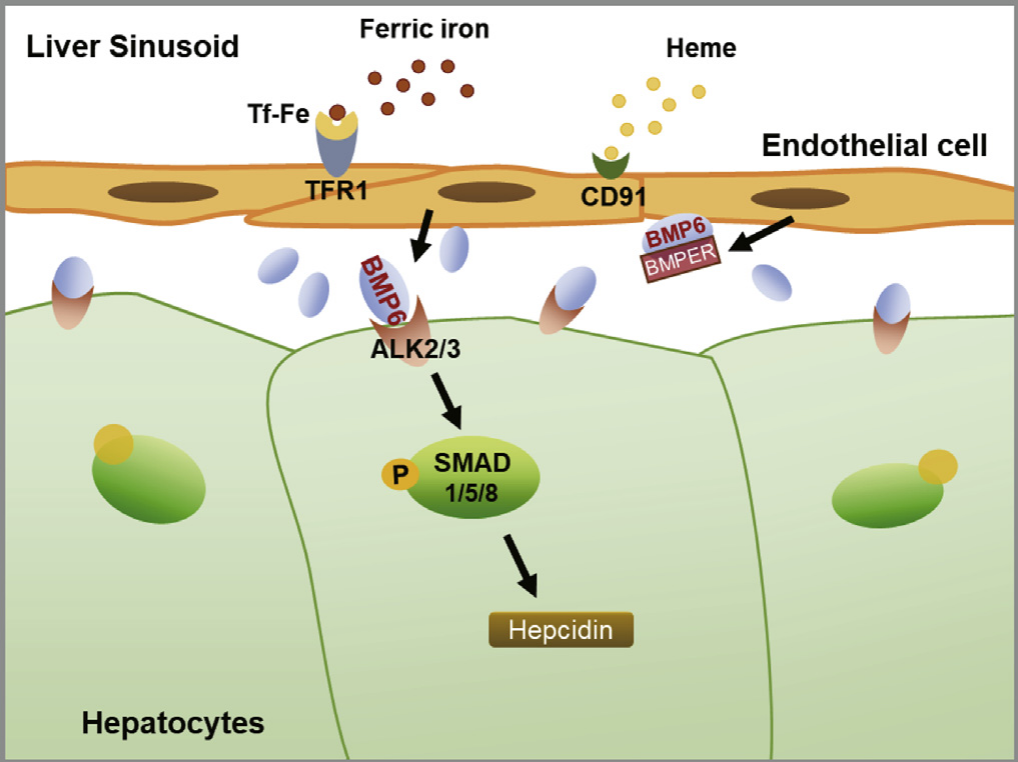
**Crosstalk between endothelial cells and hepatocytes in iron regulation.** Liver sinusoidal endothelial cells (LSECs) recognize surrounding ferric iron or heme and moderately deliver iron signaling to hepatocytes by modifying BMP6 and BMPER secretion. BMP6 can bind to ALK2/3 on hepatocytes to activate the downstream SMAD–hepcidin pathway. ALK, ALK receptor tyrosine kinase; BMP6, Bone morphogenetic protein 6; BMPER, BMP-binding endothelial regulator; SMAD, small mothers against decapentaplegic homolog.

A summarizing scheme of our findings is depicted in Figure 6. LSECs recognize surrounding transferrin-bound iron but also forms of iron such as hemin, which results in secretion of BMP6. Through direct intercellular crosstalk, BMP6 induces hepatocyte hepcidin by binding to BMP receptors (ALK2/3) on Huh7 cells to activate the downstream SMAD pathway. In addition, comparable with other hormonal loops, BMP6-antagonist BMPER is likewise but subsequently induced adjusting EC-derived BMP6 secretion in an autocrine fashion. Our data demonstrate that the presence of ECs and the secretion of BMP6 suffice to at least in part recapitulate a response of hepatocellular hepcidin as observed *in vivo*. In addition, our coculture model provides an experimental tool for better dissecting the molecular and intercellular mechanisms of crosstalk between ECs and hepatocytes for iron homeostasis.

The importance of LSECs as the predominant source for BMP6 secretion and hepcidin signaling has first been observed in mice. Mice with global endothelial BMP6 KO showed hepcidin deficiency, whereas hepatocyte and macrophage BMP6 conditional KO mice exhibited no iron metabolic injuries (25). Our ECs and Huh7 cell coculture experiments confirm that not only LSEC-derived BMP6 but also general vascular EC–derived BMP6 participates in hepatic hepcidin expression at the *in vitro* level. We also measured hepcidin expression of Huh7 cells under CM from other unrelated cell lines such as PANC-1 and HEK-293, but there was no significant difference compared with the control (data were not shown). There have been earlier reports suggesting a direct role for ECs in iron homeostasis. Thus, HUVECs cultured under high glucose levels showed higher expression levels of the iron transporters DMT-1 and IREG-1 (42). In addition, using induced pluripotent stem cell–derived brain ECs as a human blood–brain barrier model, these ECs were shown to engage in iron transport and serve as a critical regulator on the human blood–brain barrier (32). As for LSECs, it was stated that only in murine LSEC–hepatocytes coculture but not in hepatocyte monoculture, the induction of hepcidin by an ALK5 inhibitor can be observed (14). Recently, Nrf2 has been suggested as a potential key molecule in mediating iron sensing and controlling BMP6 in LSECs (38). Moreover, another study demonstrated a strong dependence of BMP6 expression in murine ECs in response to iron in the absence of serum (36). We also noticed that BMP6 expression in HUVECs was stronger modulated in the presence of low serum (2% *versus* 10% FCS) (data not shown). Additional future studies are needed to better dissect the role of serum on BMP6 expression and, for example, the role of serum transferrin. Nevertheless, our results and these supporting studies indicate that ECs are highly sensitive to iron changes in surroundings, and they are essential for iron regulation in an EC-Huh7 coculture model.

Although the BMP family consists of various members such as BMP2, BMP4, BMP6, and BMP9, not all BMPs are regulated by iron at the mRNA level as is the case for BMP6 (13). BMP6-KO mice have low levels of pSMAD1/5/8 despite severe iron overload, and their hepcidin expression is markedly reduced (30, 43). In primary murine hepatocytes, HJV deficiency severely impaired BMP6–SMAD signaling, leading to almost complete hepcidin silencing, but it did not affect the STAT3 pathway, while only slightly inhibiting BMP2–SMAD signaling (44). This latter study underlines the fact that the response of hepatocyte hepcidin to inflammation requires at least some minimal basal BMP6–SMAD signaling. Despite the significant role of BMP6, there are also some reports suggesting an independent role for EC-derived BMP2 in regulating hepcidin (45, 46). In our experimental setup, we did not see any significant changes of BMP2 transcription in HUVECs under various iron conditions (Fig. S4). However, more silencing studies of BMP2 and other BMPs should be performed in ECs in the future for a complete understanding of the distinct functions of BMPs in ECs.

Although we believe that, in contrast to *in vivo* models, *in vitro* studies are essential to better understand specific signaling interactions, there are limitations to be considered that should be addressed in future studies. Thus, we observed some differences between the two endothelial cell lines, and it remains open whether endothelial-related hepcidin signaling is restricted to LSECs or can be executed by all ECs. HUVECs are human-derived primary ECs representing the global ECs from the vascular bed, but they are not specific for the liver. In contrast, SK hep cells are generally used to mimic LSECs, but controversies continue about their origin. For example, in a recent report, SK hep cells were suggested to be a cancer stem cell of nonepithelial origin (47). In our experiments, SK hep cells were more sensitive to iron toxicity than HUVECs (Fig. S5, *A* and *B*). In addition, SK hep–CM fully activated SMAD signaling, whereas HUVECs manifested minor effects on SMAD signaling as shown in Figure 2. However, the particular inhibitors of the SMAD pathway such as heparin and epidermal growth factor in ECGM could be the reason for the blunted response in Huh7 cells under HUVEC-CM. In addition, we failed to induce BMP6 mRNA in SK hep by different concentrations of FAC (data not shown). The specific role of LSECs would require the challenge of isolating primary LSECs and coculturing them together with primary hepatocytes in response to iron changes.

We finally noted that ECs were significantly more sensitive to iron concentrations than Huh7 cells. For instance, and in contrast to a report by others (48), we observed cell growth inhibition of HUVECs starting from 10 μM hemin, whereas no toxicity was observed up to 250 μM FAC. In contrast, SK hep cells showed growth inhibition already below 10 μM for both hemin and FAC. Notably, Huh7 cells tolerate all iron forms up to maximum concentrations of 250 μM. The specific toxicity of ECs to iron has been reported earlier (49, 50). It could be an additional hint that the release of heme during hemolysis could be a critical physiological scenario for the continued basal expression of hepcidin.

In conclusion, we here establish coculture models of ECs and hepatocytes that recapitulates the fundamental *in vivo* response of hepcidin *in vitro*. Our data indicate that EC-derived BMP6 is essential for the basal expression of hepatocellular hepcidin and responds to surrounding changes of even small amounts of iron. We hope that the coculture of ECs and hepatocytes will help dissect further the complex interaction between these cells *in vivo* and their role on hepcidin signaling to develop future targeted therapies.

## Experimental procedures

### Chemicals and reagents

FAC in powder and hemin from bovine was bought from Sigma-Aldrich, and commercial SFG was obtained from the pharmacy. Salicylaldehyde isonicotinoyl hydrazone was a kind gift of Dr P. Ponka (McGill University, Montreal, Canada) (51). Human recombinant BMP6 protein and BMPER were purchased from R&D Systems (507-BP-020, 1956-CV). LDN193189 (SML0559) and methylthiazolyldiphenyl-tetrazolium bromide (MTT, M2128) powder were purchased from Sigma.

### Cell culture

Human hepatoma Huh7 cells were from the Japanese Cancer Research Resources Bank and cultured under standards conditions in DMEM (Sigma-Aldrich) with 25 mM glucose and 10% FCS, unless otherwise stated. HUVECs were purchased from the American Type Culture Collection and cultured by ECGM containing 2% FCS (PromoCell GmbH). SK hep cells were cultured with low-glucose DMEM (Sigma-Aldrich) with 10% FCS or 2% FCS. Every single experiment on cells was repeated two or three times with at least triplicates.

### Murine primary hepatocytes

Murine primary hepatocytes kindly provided by Dr Sai Wang (University of Heidelberg, Germany) were cultured under standard conditions using Williams' medium (Sigma-Aldrich). The isolation of murine primary hepatocytes followed a two-step collagenase (C2-22, Merck Biochrom) perfusion method as described previously (52). Freshly prepared hepatocytes were seeded and harvested within 48 h after isolation.

### Coculture systems

A two-dimensional coculture system of HUVECs and Huh7 together was established by seeding 5 × 10^4^ Huh7 cells per well in 12-well plates first, and after 24 h, seeding HUVECs 5 × 10^4^ on top of Huh7 cells. Similarly, SK hep and Huh7 were also kept in coculture, seeding Huh7 cells 5 × 10^4^/well first in monolayer and subsequently the same number of SK heps on top after 24 h. In the control wells of direct coculture systems, 5 × 10^4^ Huh7 cells per well were used. CM were obtained from HUVECs or SK hep monolayer culture after 24 h. Fresh ECGM or DMEM with 2% FCS served as control media for Huh7 cells under EC-CM.

### Animal experiments

Eight-week-old male mice with C57BL/6 background were obtained from Charles River Laboratories, and all animals were hosted in single ventilated cages in a 12-h day/night cycle under specific pathogen-free conditions. This animal experiment was approved by the animal ethics committee in Baden-Württemberg. SFG was diluted in sterile saline, and three mice were intravenously injected with a dosage of 625 μg in 100 μl saline. Three control animals received the same volume of saline. All mice were sacrificed after 12 h. Blood parameters and serum iron were measured by a scil Vet abc Plus device (scil animal care company GmbH).

### siRNA transfection

BMP6 siRNA transfection assay was performed on HUVECs and SK heps. HUVECs were seeded as 1 × 10^5^ in 12-well plates, and SK heps were seeded as 8 × 10^4^ 24 h before the experiment. The density of cells per well was around 80 to 90%. Transfection was performed under a reduced serum medium (Thermo Fisher; 31985062) with 50 nM BMP6 siRNA (Thermo Fisher; AM16708) or 50 nM nontargeting siRNA (Thermo Fisher; SIC001) as a negative control by Lipofectamine 2000 (Invitrogen; 13778030). Lipofectamine was applied as 3 μl per well. Transfection reagents were all removed after 6 h, and the transfection efficiency was detected at 48 h. After transfection for 24 h, the general density of cells in 12-well plates was around 50 to 60%. The knockdown efficiency was tested by real-time quantitative PCR (qRT-PCR) and Western blot (with BMP6 antibody targeted to human BMP6 synthetic peptide aa 400–500, Abcam; ab155963). In the coculture experiments, Huh7 cells were seeded on top of silenced HUVECs or SK heps (24 h after transfection) with fresh medium for further coculture experiments. The flow chart for BMP6 siRNA transfection and coculture is shown in Fig. S6.

### Western blotting, qRT-PCR, and ELISA

Cells or tissues were harvested in RIPA buffer plus 1× cOmplete protease inhibitor with EDTA (Roche Applied Sciences) on ice. Western blotting was performed as described previously (16). Primary and secondary antibodies are listed in Table S1. Immunoreactive bands were detected by the Odyssey CLx Imaging System. Band intensities were quantified using ImageJ for further statistical analysis.

Total RNA from cells and animal tissues was isolated by the TRIzol reagent (Thermo Fisher; 15596018) according to the supplier's standard protocol. Reverse transcription was performed as reported previously (16). Primers and specific probes were synthesized at Eurofins MWG Operon. Primers were designed by the Universal Probe Library (Life Science, Roche Molecular Systems), and the UPL probes were also purchased from Roche. The sequences of each primer are shown in Table S2. qRT-PCR was performed and read by the LightCycler PCR system (Roche Molecular Systems, Inc).

High binding surface microplates for ELISA were purchased from Corning Company (Product No. 9018), and a direct ELISA assay was established by human BMP6 antibody (Novus Biologicals Company, DY507) and recombinant BMP6 protein (R&D Systems, 507-BP-020). ELISA was performed according to the manufacturer's protocol.

### Statistical analysis

All data are shown as the mean ± SD. Significant differences (n.s., no significance; ∗*p* < 0.05; ∗∗*p* < 0.01; ∗∗∗*p* < 0.001) between means of datasets were assessed by one-way ANOVA with Tukey's test or unpaired two-tailed Student's *t* test using GraphPad Prism 6 software.

## Data availability

All data are within the article and supporting information. Any additional information or data are available upon request.

## Supporting information

This article contains supporting information.

## Conflicts of interest

The authors declare that they have no conflicts of interest with the contents of this article.
