# Supplementary material for: Bone morphogenetic protein 6–mediated crosstalk between endothelial cells and hepatocytes recapitulates the iron-sensing pathway in vitro
Source: J Biol Chem. 2021 Nov 2;297(6):101378. doi: 10.1016/j.jbc.2021.101378 (PMC8637636; doi:10.1016/j.jbc.2021.101378)
Supplement: Supplemental Figures S1–S6 and Tables S1, S2 [file mmc1.docx]

**Bone morphogenetic protein 6-mediated crosstalk between endothelial cells and**

**hepatocytes recapitulates the iron sensing pathway *in vitro***

**Supplementary Tables**

**Suppl Tab. 1 Antibodies and dilutions**

| **Antigen** | **Host Species** | **Dilution** | **Company** | **Cat. No.** |
| --- | --- | --- | --- | --- |
| GADPH | rabbit | 1:3000 | CST | 2118s |
| TFR1 | mouse | 1:1000 | Invitrogen | 13-6800 |
| BMP6 | mouse | 1:1000 | Novus | MAB507 |
| BMP6 | rabbit | 1:1000 | Abcam | ab155963 |
| pSMAD1/5/8 | rabbit | 1:1000 | CST | 13820 |
| SMAD1 | rabbit | 1:1000 | CST | 9743 |
| Rabbit Ig | goat | 1:10000 | LI-COR | 926-32210 |
| Mouse Ig | Goat | 1:10000 | LI-COR | 926-32211 |

**Suppl Tab. 2 Primers list of quantitative RT-PCR**

| **Gene** | **Primer sequence** |
| --- | --- |
| **Human β2MG** | forward: 5’-tga ctt tgt cac agc cca aga ta-3' |
|  | reverse: 5’-aat cca aat gcg gca tct tc-3' |
|  | probe: FAM-tga tgc tgc tta cat gtc tcg atc cca-TAM |
| **Human Hepcidin** | forward 5’-cag gac aga gct gga gcc a -3' |
|  | reverse: 5`-gca gca cat ccc aca ctt tg-3` |
|  | probe: FAM-ctg ctg cgg ctg ctg tca tcg a-TAM |
| **Human BMP6** | forward: aca tgg tca tga gct ttg tga |
|  | reverse: act ctt tgt ggt gtc gct ga |
|  | probe UPL #22 (Roche) |
| **Human BMPER** | forward: gca act aca atg gac ata aac gtg |
|  | reverse: cag caa agt cat cca cat caa |
|  | probe UPL #22 (Roche) |
| **Human HO-1** | forward: aac ttt cag aag ggc cag gt |
|  | reverse: ctg ggc tct cct tgt tgc |
|  | probe UPL #63 (Roche) |
| **Human SMAD6** | forward: tgc aac ccc tac cac ttc a |
|  | reverse: cga gga gac agc cga gag t |
|  | probe UPL #10 (Roche) |
| **Human SMAD7** | forward: aaa cag ggg gaa cga att atc |
|  | reverse: acc acg cac cag tgt gac |
|  | probe UPL #50 (Roche) |
| **Human Id1** | forward: aaa cgt gct gct cta cga ca |
|  | reverse: gga acg cat gcc gcc t |
| **Mouse HPRT** | forward: ggt cca ttc cta tga ctg tag att tt |
|  | reverse: caa tca aga cgt tct ttc cag tt |
|  | probe UPL #22 (Roche) |
| **Mouse Hepcidin** | forward: gat ggc act cag cac tcg |
|  | reverse: ctg cag ctc tgt agt ctg tct ca |
|  | probe UPL #63 (Roche) |
| **Mouse BMP6** | forward: cac agt cct ctt ctt cgg gc |
|  | reverse: ctt ttg cat ctc ccg ctt ct |
|  | Probe: cct cta tcg gcg gct caa gac cca |
| **Mouse BMPER** | forward: cgt ctt gct gct gct caa t |
|  | reverse: acc tcc cct tca ttt tca ca |
|  | probe UPL #12 (Roche) |

**Supplementary Figures**

**Suppl. Fig. 1**


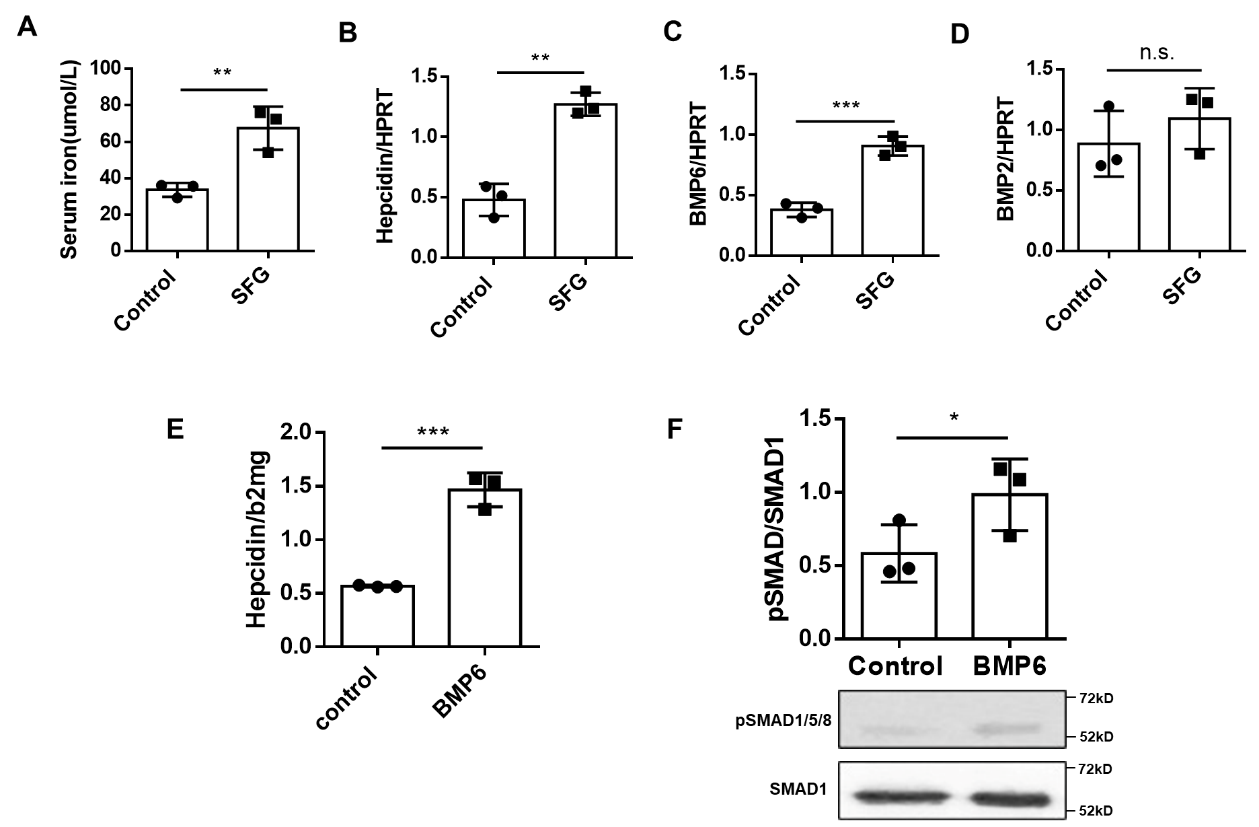


**Suppl. Fig. 1 BMP6/SMAD pathway contributes to hepcidin expression both in vivo and in vitro.** A) Intravenous injected with 625 μg SFG (sodium ferric gluconate) in 100 μl volume or the same volume of saline and sacrificed after 12 hours, mice with SFG gained more serum iron. B) Mice with iron overload had higher hepcidin mRNA expression in the liver (3 mice in each group). C and D) Iron overloading increased BMP6 mRNA expression but not BMP2 expression in mice liver. E) Hepcidin expression in Huh7 cells was efficiently enhanced under the treatment of 50 ng/ml human recombinant BMP6 protein for 24 hours. Meanwhile, pSMAD1/5/8 protein appeared higher expression than the control cells. Huh7 cells were cultured under Dulbecco's modified Eagle medium (DMEM) with 10% fetal calf serum. Target proteins expression was determined by Western Blot. Representative data for three independent experiments are used for protein statistical analysis. Hepcidin and BMP6 mRNA were determined by quantitative reverse transcription PCR (qRT-PCR). PCR results were normalized to HPRT or β2MG. Data are presented as dot plots with mean ± SD and significant differences are marked by asterisks (n.s., not significant; *, p < 0.05; **, p < 0.01; ***, p < 0.001).

**Suppl. Fig. 2**


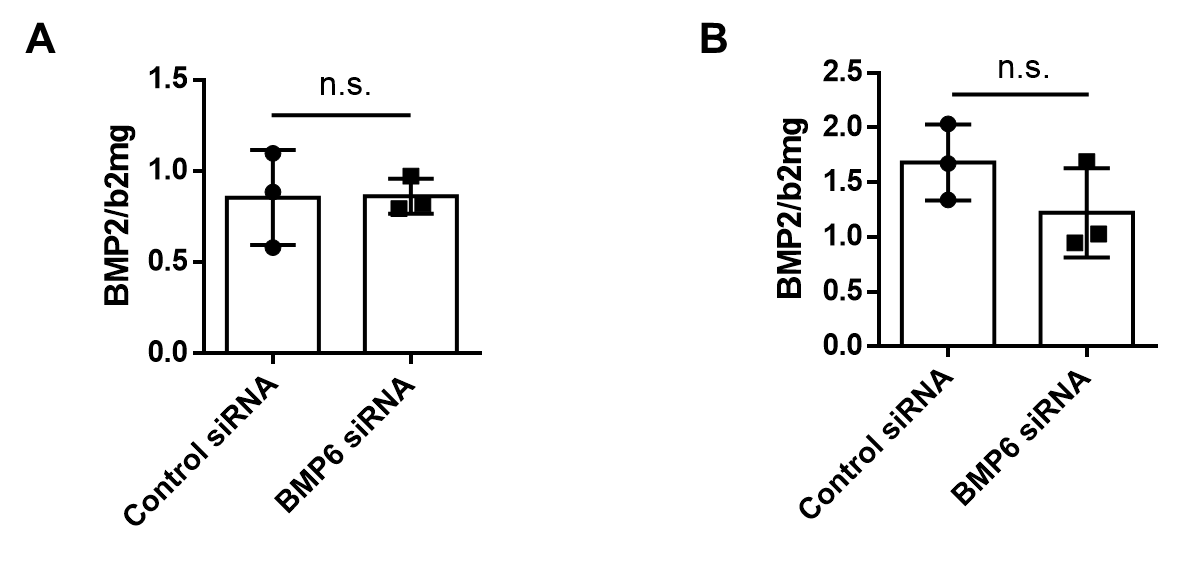


**Suppl Fig. 2 BMP6 siRNA has no effect on BMP2 transcription levels.** A) BMP2 mRNA expression was not changed by BMP6 siRNA transfection in HUVECs. B) BMP2 mRNA expression was not changed by BMP6 siRNA transfection in SK heps. BMP2 mRNA were determined by quantitative reverse transcription PCR (qRT-PCR). Representative data for three independent experiments are shown. PCR results were normalized to β2MG. Data are presented as dot plots with mean ± SD and significant differences are marked by asterisks (n.s., not significant).

**Suppl. Fig. 3**

**Suppl. Fig. 3 The upregulation of Id1 mRNA expression by SK-hep conditioned medium is inhibited by LDN.** Id1 mRNA were determined by quantitative reverse transcription PCR (qRT-PCR). PCR results were normalized to β2MG. Data are presented as dot plots with mean ± SD and significant differences are marked by asterisks (n.s., not significant; *, p < 0.05; **, p < 0.01).

**Suppl. Fig. 4**

**Suppl. Fig. 4 BMP2 expression in HUVECs under ferric ammonium citrate.** BMP2 mRNA expression in HUVECs was not changed under different concentrations of ferric ammonium citrate (FAC 0.5 μM, 5 μM, 50 μM in endothelial cell growth medium). BMP2 mRNA was determined by quantitative reverse transcription PCR (qRT-PCR). PCR results were normalized to β2MG. Data are presented as mean ± SD and significant differences are marked by asterisks (n.s., not significant).

**Suppl. Fig. 5**


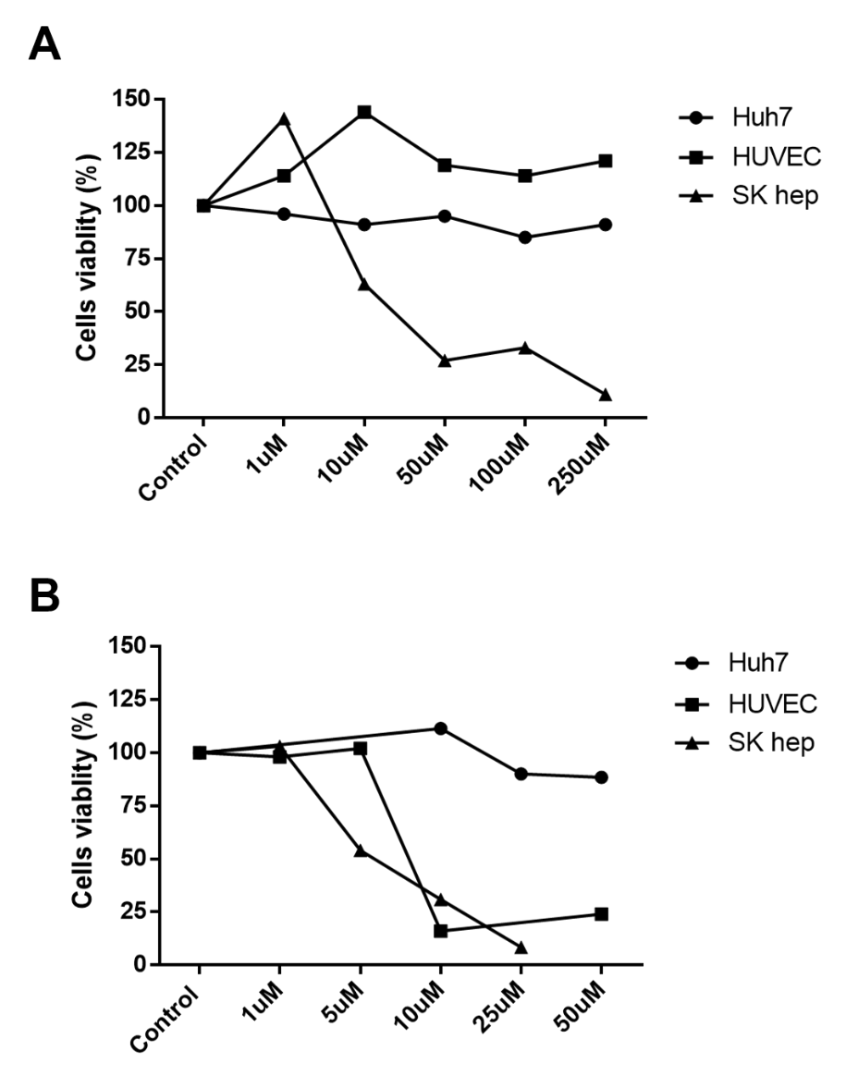


**Suppl. Fig. 5 Cell viability under gradient concentrations of FAC or hemin for 24 hours A)** Huh7 cells, HUVECs and SK hep were separately treated by FAC 1 μM, 10 μM, 50 μM, 100 μM and 250 μM. In the cell viability curves, SK hep was the most sensitive cell line to FAC concentrations. 10 μM FAC started to be toxic to SK hep. Its viability decreased to 60% under 10 μM FAC, even as low as 30% under 50μM FAC. These gradient concentrations of FAC had no significant influence on HUVECs and Huh7 cells' viabilities. **B)** Huh7 cells, HUVECs and SK hep were separately treated by hemin 1 μM, 5 μM, 10 μM, 25 μM and 50 μM. Hemin 5uM was already toxic to SK hep and the cell viability dropped to around 50%. Similarly, hemin over 5uM inhibited HUVECs growth and their cell viability drastically decreased to 20% under 10μM hemin. These gradient concentrations of hemin had no significant influence on Huh7 cells viability. HUVECs were cultured by endothelial cell growth medium (ECGM) containing 2% fetal calf serum (FCS); SK heps were cultured by Dulbecco's modified Eagle medium (DMEM) with 2% FCS; and Huh7 cells were cultured by DMEM with 10% FCS. These cell viabilities were determined by MTT assay.

**Suppl. Fig. 6**


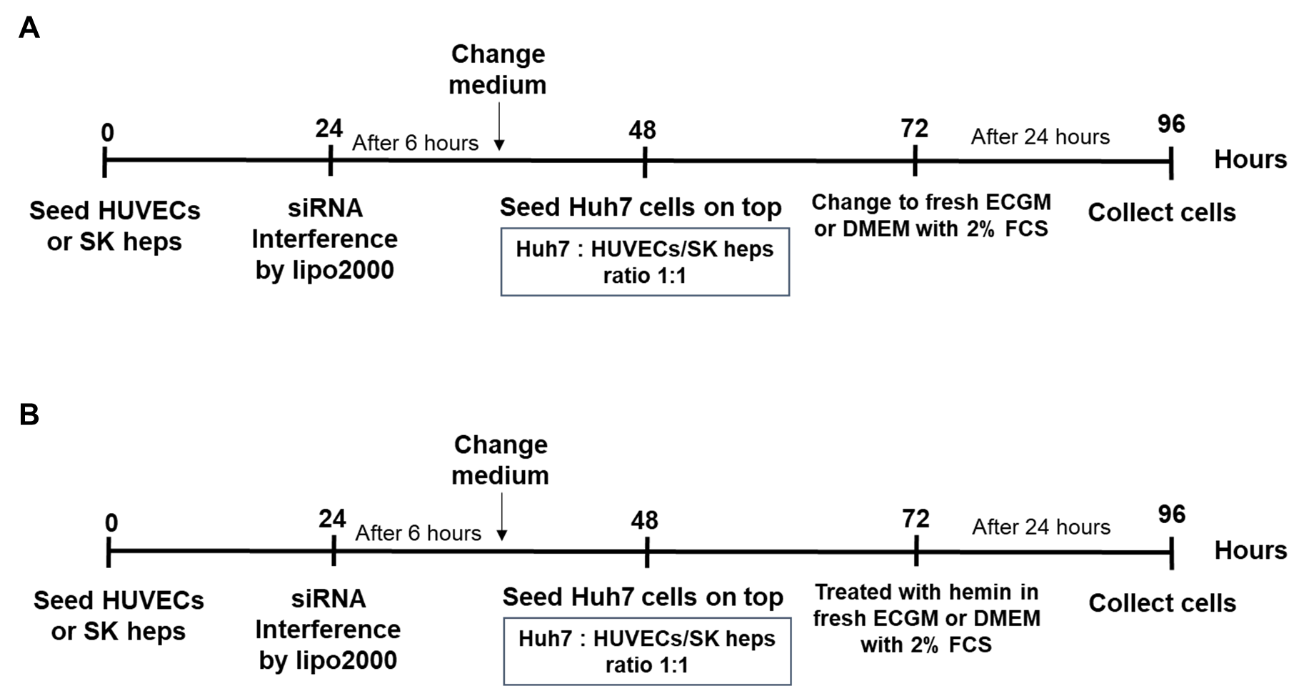


**Suppl. Fig. 6 Procedures of BMP6 siRNA transfection in endothelial cells and hepatocytes co-culture without treatment (A) or with hemin treatment (B).**
